# Understanding Vietnamese Urban Consumers’ Nutrition Label Use, Health Concerns, and Consumption of Food and Beverages with Added Sugars

**DOI:** 10.3390/nu12113335

**Published:** 2020-10-29

**Authors:** Duc Nguyen-Anh, Wendy J. Umberger, Di Zeng

**Affiliations:** 1 The Centre for Global Food and Resources, The University of Adelaide, 10 Pulteney St, Adelaide, South Australia 5000, Australia; anhduc.nguyen@adelaide.edu.au (D.N.-A); di.zeng@adelaide.edu.au (D.Z.); 2 Department of Quantitative Analysis, Faculty of Economics and Rural Development, Vietnam National University of Agriculture, Hanoi 10000, Vietnam

**Keywords:** nutrition label, health concern, added sugars, consumption, urban, food diary, dietary transition, Vietnam

## Abstract

Vietnam is experiencing a diet and nutrition transition. Increasing consumption of food and beverages with added sugars is a significant public health concern. Policies and interventions, such as mandatory nutrition labelling, are being considered to improve consumers’ awareness and understanding of diet and health implications of added sugars in food and beverages. The effectiveness of various policy approaches relies on an improved understanding of the interrelationships between urban Vietnamese consumers’ health concerns, nutrition label use, and intake of sugars. We empirically disentangle these relationships for urban Vietnamese households using novel intra-household data covering 4047 adults and 737 adolescents from 1590 households in Hanoi and Ho Chi Minh City. The data are from comprehensive household surveys and 24-hour food diaries. Simultaneous equation regression models are estimated using three-stage least squares (3SLS) to account for possible endogeneity. Nutrition label use is significantly associated with a lower share of calories from foods and beverages with added sugars. These findings suggest that nutrition labelling programs may be an effective policy mechanism to reduce the negative health implications of increasing availability and consumption of food and beverages with added sugars in urban Vietnam.

## 1. Introduction

Changing food systems, diet transition resulting in increasing consumption of highly processed non-traditional foods, and more sedentary lifestyles are all contributing to the growing prevalence of non-communicable diseases (NCDs), such as cardiovascular disease, obesity, type 2 diabetes, and some types of cancer, in emerging and developing economies across the Southeast Asia region [1,2,3,4,5].

One particular concern resulting from the diet transition occurring in many countries is increasing consumption of non-traditional foods and beverages with added sugars [6,7,8,9]. Added sugars are defined as all sugars and syrups (monosaccharides and disaccharides) eaten separately or used as ingredients in processed or prepared foods [10]. Higher consumption of foods and beverages with added sugars [10] has been shown to be associated with excess energy intake and poorer diet quality [11,12], resulting in a heightened risk of NCDs, particularly overweight and obesity, and type II diabetes [13,14].

Increasing consumption of food and beverages with added sugars is now a significant public health concern in Vietnam [15,16]. In fact, per capita availability of sugar and sweets in Vietnam increased 176% from 1961 to 2013 [4]. Total sugar consumption per capita in Vietnam (estimated at 46.5 g per person per day) is nearly twice the maximum level (25 g per person per day) recommended by the World Health Organization (WHO) [17,18].

The prevalence of overweight and obesity in urban Vietnam has increased rapidly among all age groups. An estimated 23% and 17% of Vietnamese adult females and males, respectively, were estimated to be overweight or obese in 2016, up from 13% and 8%, respectively, in 2000 [4,19]. The same report estimated that, although only about 2% of Vietnamese children and adolescents (aged five to 19) were overweight or obese in 2000 [19], by the year 2016, the prevalence of overweight and obesity had increased dramatically, with the prevalence for boys (15%) being much higher than for girls (9%) [19]. This increased prevalence of overweight and obesity in Vietnamese children and adolescents is especially concerning because the associated risk of other diet-related NCDs is subsequently higher in later life stages [15,20,21]. Furthermore, the incidence of diabetes is growing at alarming rates and has almost doubled from 3.7% in 2004 to 6% in 2016 [22,23]. In 2017, the annual average cost per patient with diabetes in Vietnam was estimated to be US$246, or an estimated 12% of the GDP per capita [24].

The growing prevalence of overweight and obesity and diabetes in urban Vietnam, combined with rising per capita consumption of food and beverages with added sugars (e.g., highly processed breads, cakes, sugar-sweetened dairy and fruit drinks and carbonated beverages), suggest that government interventions may be needed to avoid longer-run negative impacts on human health and productivity as Vietnam continues to develop and urbanize.

Some policy options that have been considered include imposing an excise tax on sugar-sweetened beverages, raising consumer awareness about the harmful effects of food with high amounts of added sugars, and mandatory nutrition labels on food products [16]. In Vietnam, nutrition labelling regulations state that food companies must provide information about sugar content (e.g., grams) on pre-packaged foods. However, labelling specific information about the amount of added sugars in foods is currently not required; and generally, it is not available for consumers at the point-of-purchase [25]. Understanding the potential effectiveness of these current regulations and other policy options relies on an improved knowledge of the interrelationships between consumers’ use of nutrition labels, health concerns, and consumption of added sugars.

Food labels that include nutrition information (e.g., nutrition panels) have been shown to assist consumers in making healthier food choices [26,27,28]. Several studies have found that individuals who use nutrition labels tend to have healthier diets as measured by Healthy Eating Index (HEI) scores [29,30,31]. A number of studies in high-income countries reported a significant correlation between nutrition label use and individuals’ dietary intake [28,32]. For example, label use was associated with higher fiber and iron intakes [33], lower energy intakes from fat, carbohydrates, and sodium [34,35,36,37], and a significant reduction in the share of energy intake from added sugar [38].

Most previous studies have not explicitly considered the role of health concerns, a latent factor that could affect both the use of nutrition labels, and dietary intake of certain nutrients such as added sugars [32,38,39,40,41]. Yet, consumers’ health concerns may have an indirect relationship with dietary intake of food and nutrients through food label use [39,40]. Consumers who are more concerned about nutrition and health have been found to be more likely to use nutrition labels [32]. Consumers may search for nutrition information because they want to avoid potential negative effects of consuming high amounts of certain nutrients such as saturated fat, salt, and added sugars [40,41]. This could also explain why consumers who looked for specific nutrient information when purchasing food tended to reduce their energy intake by minimizing consumption of foods high in some of these nutrients (e.g., fat and sugar) [38,42].

In addition to food label use and nutrition and health concerns, previous studies have found several sociodemographic factors (e.g., age, education, gender, income) to be associated with intake of food and beverages with added sugars [38,43]. For example, a study of over 9000 adults from eight Latin American countries [43] found that females and younger individuals had a higher share of energy intake from foods with added sugars as compared to men and older age groups. Other studies conducted in Southeast Asia found that households with older respondents had a lower share of healthy food [44], or a lower intake of vitamin A and protein [45]. Household income was found to have a nonlinear relationship with individuals’ daily caloric intake from macronutrients [46,47].

While previous research has examined the impact of label use on individuals’ dietary intake [38,39,40,41,42], little is currently known about how consumers’ health concerns may influence their dietary intake. A few studies exploring this hypothesized relationship exist, but these studies are based on a sample of adolescents and university students in other countries, and are not likely to represent the Vietnamese population [48,49]. Moreover, these previous studies did not account for possible linkages between consumers’ health concerns and label use with respect to dietary intake of sugar.

Therefore, this study aims to understand the relationship between Vietnamese urban households’ health concerns related to added sugar, their use of nutrition labels, and individual household members’ energy intake from foods and beverages with added sugars. Our main objectives are to: (1) Understand the relationship between household-level use of nutrition labels and the share of daily calories from foods with added sugars for adults and adolescents living in the household [38,39,40,41,42]; (2) understand the association between household-level concerns about sugar content in food and the share of daily calories from foods with added sugars [39,40]; and (3) understand the relationship between household-level concerns about added sugar in food and use of nutrition labels [32].

Two main hypotheses are investigated: (1) Household-level use of nutrition labels and concerns about added sugar in foods jointly affect the share of calories from foods with added sugars for adults and adolescents living in the household [32,38,39,40,41,42]; (2) households’ concerns about added sugar directly affect households’ use of nutritional labels [32].

The contributions of this study are three-fold. First, we not only consider the relationship of nutrition label use on individuals’ dietary intake of sugars, but we also analyze how concerns about intake of added sugar affect the use of nutrition labels and dietary intake of added sugars. This understanding allows us to provide broader insight on the mechanisms affecting individuals’ consumption behavior with respect to intake of sugary foods and beverages. Second, while similar studies have focused on high-income countries such as the United States or Europe, this is the first study to investigate the impact of nutrition label use on dietary intake in Vietnam, where such knowledge is urgently needed because the country is at the intermediate stage of diet transition [6] and is considering potential policy interventions to improve diet quality. Third, a subsample analysis is conducted for different gender and age groups to capture the possible heterogenous effects of nutrition label use and concerns related to sugar on individuals’ nutrition outcomes, thereby providing information useful for developing policies to target segments of the population most at-risk of developing obesity or diabetes.

The remainder of this paper is structured as follows. Section 2 describes the data and explains the empirical methods of the following analysis. Section 3 discusses the empirical results. Section 4 concludes with policy implications that arise from our findings.

## 2. Materials and Methods

### 2.1. Survey of Households and Individuals

The data used in this study were derived from a comprehensive household study (conducted by the authors) covering 1590 households in Hanoi and Ho Chi Minh City, Vietnam. In the study, individual food consumption information for 4047 adults and 737 adolescents (aged between 10 to 19 years of age) was collected using 24-h food diaries.

The household survey was implemented between December 2016 and March 2017 (with a four-week break to avoid food consumption fluctuations around Tet, the Vietnamese lunar New Year). Households were selected using a proportional random sampling strategy, where the probability of selecting each ward (the primary sampling unit within each city) is proportional to its population. The survey was conducted by trained enumerators through face-to-face interviews with the individual in the household who was responsible for household food purchasing and cooking activities (referred to hereafter as the “household respondent”). Data were collected on Android tablets with a customized data application developed by CommCare, a major mobile data collection platform that prevented missing entries of key information and included the GPS coordinates of households.

The household questionnaire was adapted from an instrument used for a similar study conducted in Indonesia [8]. The questionnaire was used to collect the following information at the household-level: food expenditures for 92 different food and beverage products; the type of food outlet where food products are typically purchased; access to various types of food outlets (physical distance and travel time to nearest outlet); non-food expenditures; use of food ingredient and nutrition labels when shopping for food, and if labels are used, what type of label information they consider. A set of questions was also included to understand household nutrition attitudes and concerns about the content of sugar, fat, cholesterol, and salt in food. Sociodemographic information was collected for each individual living in the household (including their role in the household).

The 24-hour food diaries were used to collect detailed food and beverage intake data for each individual living in the households for four different days throughout 2017. Data were collected on multiple days throughout the year to account for seasonal variation in individuals’ food consumption behavior. Food and nutrient intake data for a total of 786 food items was computed for each individual in the household [50].

Quantities of each food item reported for an individual (i) were converted into gram equivalence for all food items using the FAO INFOODS databases [51]. The daily intake for each food item was calculated using a weighted average method. For example, if an individual i consumed m (grams) of rice in n days (n≤4), then his/her daily intake for rice was m/n (grams). After calculating the weight equivalents, single food items were converted into macronutrients (in grams) using the 2007 Vietnamese Food Composition Table (VFCT), and the updated online version of the 2017 VFCT [52]. The nutrient contents of mixed dishes that were not included in the VFCT database were calculated by identifying the average component ingredients from Vietnamese recipes [53]. The total grams of each macronutrient were summed, and the Atwater coefficients (kcal/g) were used to convert the gram equivalent of each macronutrient to calories (i.e., 4kcal/g for carbohydrates and protein, and 9 kcal/g for fat) [54]. The mean daily caloric intake was computed by summing the calorie contributions of all food items. We exclude food diary data for children less than 10 years of age because school-age children in urban Vietnam usually eat lunch at school during the week [55].

### 2.2. Measuring Added Sugar Intake

The dependent variable in the estimated model, $SugarCalorie_{ihc}$ represents, for individual i, living in household h, located in city c, the share of daily caloric intake from food and beverages with added sugars. Based on similar studies in Asia [56,57], and after consulting with nutrition scientists at the National Institute of Nutrition in Vietnam, a list of food and beverages with added sugars was created. The full list is provided in the Appendix A (Table A1).

$SugarCalorie_{ihc}$ was constructed by dividing individual *i*’s mean daily caloric intake from food and beverage products with added sugars by their mean total daily caloric intake (Table 1). This measure has been used by a number of studies conducted across the United States, Europe, and Malaysia [11,56,58]. It allows us to compare individuals with different caloric needs and to understand which individuals consumed a relatively larger proportion of their daily caloric intake from food and beverages with added sugars [38].

### 2.3. Variables Explaining Sugary Food and Beverage Consumption

#### 2.3.1. Main Explanatory Variables

Two key explanatory variables are considered in the analyses: (1)$LabelSugar_{hc}$ and (2) $AvoidSugar_{hc}.$ Both of these variables were captured at the household-level (h). Information was provided by the household respondent who was the person primarily responsible for making household food purchase decisions. In most cases, the respondent was also responsible for preparing meals for the household.

In the survey, household respondents were first asked to indicate how often (1 = always, 2 = often, 3 = sometimes and 4 = never) they used food ingredient and nutrition labels when shopping for food. If a respondent indicated that they used food labels at least “sometimes” then they were asked a follow-up question: “What type of nutritional information do you use or look for?” They were provided with a list of nine possible options which included sugar, calories, salt/sodium, fat and an “other” option. $LabelSugar_{hc}$ is a binary variable equal to 1 if the respondent for household h indicated they searched for information on sugar content when shopping for food (Table 1).

The variable $AvoidSugar_{hc}$ is a proxy for concerns about added sugar in food and beverages. The variable is based on the household respondents’ answers to a question where they were asked to indicate how strongly they agree or disagree (1 = strongly disagree and 5 = strongly agree) with the following statement: “I avoid purchasing food and drinks with a high amount of sugar.” As we wanted to identify those household respondents that consciously avoided purchasing food and drinks with high amounts of sugar, we dichotomized the original five-point scale so that $AvoidSugar_{hc}$ equals 1 if the household respondent indicated they “somewhat agree/strongly agree” and equals to 0 if they indicated they were “unsure/somewhat disagree/ strongly disagree” (Table 1). Only a few (n = 21) respondents indicated that they were “unsure” when answering this question. Alternative grouping of the 21 “unsure” respondents into the opposite category yielded almost identical regression results (given their limited number).

#### 2.3.2. Other Individual and Household Covariates

Characteristics of both the household respondent and the individual (i) may influence our main outcome variable: $SugarCalorie_{ihc}$. Following the literature [38,43,44,45], two categorial variables are incorporated to indicate individual i’s age (Age) and the household respondent’s age (RespAge) [37,59]. Additionally, the binary variable Female (equal to 1 if the individual was female) is included to indicate gender [55]. We also include the respondent’s education level (RespEduc) measured in the number of years of school completed (Table 1).

Other household characteristics have been shown to have significant impacts on individuals’ dietary intake [8,42,46,47,50,60] (Table 1). Thus, we include both Income and $Income^{2}$ to capture the possible non-linear relationship between household income and the share of total calories from added sugars [58,59]. Three dummy variables are used to capture the effect of having at least one child in the household aged from under five years (Child_under5), five to nine years (Child_5to9), and 10 to 19 years (Child_10to19) [8]. A dummy variable (Smoker) representing the presence of any cigarette smoker in the family is also included in the model to capture possible effects of an unhealthy lifestyle on individuals’ diet quality [42,60]. Finally, a city dummy variable (Hanoi) is used to control for unobservable city-level effects (e.g., social norms, cultural traditions, dietary patterns and levels of economic development) that may differ between cities and affect the outcome variable [8].

### 2.4. Statistical Analysis

We hypothesize that the intake of food and beverage products with added sugars is associated with both household respondents’ use of nutrition labels (LabelSugar) and their attempts to avoid added sugars in food and beverages (AvoidSugar). We also hypothesize that the variables AvoidSugar and LabelSugar are interrelated. To examine the potential interrelationships among these variables, we estimate a simultaneous equation model specified as follows:

(1) $$\{\begin{array}{c} {\mathrm{AvoidSugar}}_{\mathrm{hc}}=\alpha_{0}+\alpha_{1}{\mathrm{LabelSugar}}_{\mathrm{hc}}+\alpha_{2}X_{\mathrm{ihc}}+\alpha_{3}Z_{1\mathrm{hc}}+e_{1\mathrm{ihc}}\\ {\mathrm{LabelSugar}}_{\mathrm{hc}}=\beta_{0}+\beta_{1}{\mathrm{AvoidSugar}}_{\mathrm{hc}}+\beta_{2}X_{\mathrm{ihc}}+\beta_{3}Z_{2\mathrm{hc}}+e_{2\mathrm{ihc}}\\ {\mathrm{SugarCalorie}}_{\mathrm{ihc}}=\gamma_{0}+\gamma_{1}{\mathrm{LabelSugar}}_{\mathrm{hc}}+\gamma_{2}{\mathrm{AvoidSugar}}_{\mathrm{hc}}+\gamma_{3}X_{\mathrm{ihc}}+e_{3\mathrm{ihc}} \end{array}$$

In the above system of equations (Equation (1)), $AvoidSugar_{hc}$ indicates that the respondent for household h agreed or strongly agreed with the statement that they avoid purchasing food and drinks with a high amount of sugar. $LabelSugar_{hc}$ indicates that the respondent uses nutrition labels (when available) to obtain information about sugar content in food products. $SugarCalorie_{ihc}$ measures individual i’s share of daily caloric intake from food and beverages with added sugars. $X_{ihc}$ is a vector of the individual and household characteristics explained earlier. $Z_{1hc}$ and $Z_{2hc}$ are two vectors of instrumental variables, and $e_{1ihc}$ to $e_{3ihc}$ are random error terms which are assumed to be independent and identically distributed in the model.

Equation (1) represents a non-recursive system of equations where the reciprocal effect exists in the first two lines of the system equations, for $AvoidSugar_{hc}$ and $LabelSugar_{hc},$ thus, they cannot be estimated with stepwise ordinary least squares. Previous studies have suggested that label use and health concern are likely to be determined by some of the factors that also affect the intake of sugars [42,59]. Therefore, in our system of equations (Equation (1)), the variables $AvoidSugar_{hc}$ and $LabelSugar_{hc}$ are used on both the left and right sides; and the error term, $e_{3ihc}$, is potentially correlated with these variables. In this case, three-stage least squares method (3SLS) is an appropriate estimation method to use since it obtains instrumental variable estimates considering the covariances across equation disturbances [61]. This method can produce consistent estimates and generalized least squares is used to account for the correlation structure in the disturbances across the equations.

We use $InfoSourceDoctor_{hc}$ and $HealthProblem_{hc}$ as instruments ($Z_{1hc})$ for the endogenous variable $AvoidSugar_{hc}$. The binary indicator variable $InfoSourceDoctor_{hc}$, indicates whether or not household members received nutrition information or advice from medical professionals (i.e., doctor, nurse, and nutritionist). The second instrument, $HealthProblem_{hc}$ reflects whether any household member had been previously diagnosed as having a chronic health problem related to high sugar consumption (e.g., overweight or obesity, diabetes, high blood pressure, or heart disease). We expect these instruments to have a high correlation with $AvoidSugar_{hc}$. Medical doctors are seen as the most important source of nutrition information and may be able to encourage individuals to follow certain dietary recommendations [62]. Their health advice may increase the respondent’s concern about sugar, but the respondent’s concern may not be directly linked with an individual’s intake of added sugars ($SugarCalorie_{ihc})$. Similarly, having a household member with chronic health problems related to sugar consumption may be associated with an individual’s intake of added sugar through increasing household respondent’s concerns about sugar.

$DistanceWetMarket_{hc}$ is used as an instrument ($Z_{2hc})$ for the endogenous variable, $LabelSugar_{hc}$. This instrument measures the distance from the individual’s home to the nearest wet market (traditional formal Vietnamese food market) [63]. We expect that the instrument is negatively associated with label use as a shorter distance to a traditional wet market may encourage consumers to buy more fresh foods which are generally more available at wet markets. Further, point-of-purchase nutrition information is not usually accessible at wet markets [63]. The instrument is not expected to be directly linked with the main outcome variable as it is unlikely to directly influence an individual’s consumption of foods with added sugar. To ensure that all instruments are not correlated with the share of calories from added sugars ($SugarCalorie_{ihc}),$ we conducted a Pearson correlation coefficient test. We found very low correlations between the dependent variable, $SugarCalorie_{ihc}$ and the instrumental variables ($DistanceWetMarket_{hc}$= 0.014; $InfoSourceDoctor_{hc}$= 0.019; and $HealthProblem_{hc}$= −0.029).

The summary statistics for all variables used in the estimations are provided in Table 1. It is worth noting that all three variables of primary interest exhibit relatively large variations from the mean. For example, the standard deviation of $LabelSugar_{hc}$(0.48) is larger than its mean (0.38), suggesting the existence of large variance across households, which is beneficial to our analyses.

## 3. Results and Discussion

### 3.1. Full Sample Analysis

Table 2 reports the 3SLS estimation results. Before interpreting individual coefficients, a non-recursive structural equation model (SEM) was first employed to test the covariance between the dependent variable and the two potentially endogenous variables. This method is similar to the Hausman endogeneity test [64]. The chi-squared test statistic of 40.99 (significant at the 1% level) provides evidence to suggest that the variables are indeed endogenous. As shown in Table 2, the coefficients on the instrumental variables: *DistanceWetMarket* (−0.021), *InfoSourceDoctor* (0.070), and *HealthProblem* (0.154) are all statistically significant at the 5% level. Thus, they appear to satisfy the relevance condition. The first-stage F-statistics (36.44 and 37.21) are both significant at the 1% level and have values greater than 10, suggesting that the instruments are likely to be valid [65]. These results give credence to the overall set of instruments used in the 3SLS estimation. 

The coefficients from the estimation of Equation (1) for the full sample of adults and adolescents are shown in Table 2. The empirical results show that nutrition label use (LabelSugar) is negatively associated with individuals’ share of calories from food and beverages with added sugars (SugarCalorie). This suggests that if the household respondent (the person in the household responsible for food purchases and preparation) generally uses food labels to access sugar content information, then individual members of the household are more likely to have a lower share of their calories from added sugars. This result is in line with other literature that suggests that nutrition label use can have significant and positive impacts on the diet quality of the household members [33,38,42]. The coefficient on AvoidSugar suggests that respondent’s attempts to avoid sugar are not statistically significant in explaining higher consumption of added sugar (SugarCalorie). One possible explanation is that although many respondents (73%) indicated that they tried to avoid food and beverages with added sugar, they might be not be aware of the main sources of added sugar, and would have no way of knowing the sugar content if relevant nutrition information is not accessible [40].

Nutrition label use is negatively associated with the variable that indicates household respondents try to avoid sugar, yet the converse relationship does not hold (Columns 2–3, Table 2). These results seem to suggest that respondents who indicate that they try to avoid sugar are not using nutrition labels, however, this could be because of limited access to nutrition labels, i.e., a large share of the food and beverage products available at markets, particularly traditional markets, do not have nutrition labelling. Thus, policies that encourage more food retailers and food processors to provide nutrition labels on products and/or point of purchase nutrition information may help consumers who are trying to avoid food and beverages with added sugars, ultimately to reduce the consumption of added sugars.

Simple statistical analysis of our data shows that on average, compared to other groups, younger individuals and females tend to have a higher share of their caloric intake from food and beverages with added sugars (Table 3). Thus, it is not surprising that the coefficients on the AgeGroup variables in Table 2 are negative and significant, indicating that adults, as compared to adolescents, consume relatively less food and beverages with added sugars. This is consistent with other literature that highlights the risk of high intake of added sugars among adolescents [6,11,55]. The coefficient on *Female* is significant and positive, thus indicating that, on average, Vietnamese females tend to consume a higher share of calories from added sugars as compared to men, which also echoes the results of other studies [38,55]. These results suggest that there are potential heterogenous effects which differ by gender and across adults and adolescents, this potential heterogeneity is further explored through the subsample analyses discussed in Section 3.2.

In the analyses of the full sample, we also find significant relationships between the household respondent’s age (RespAge) and education (RespEduc) and the individual’s share of calories from added sugars. Results indicate that individuals tend to have a relatively higher share of calories from added sugars if the age of the household respondent is between 40 and 50 years as compared to the reference group of under 30 years. Moreover, the household respondent’s education is positively associated with the share of food energy from added sugar consumed by individuals in the household. Yet, respondent’s education level is positively associated with their use of nutrition labels and avoidance of food and beverages with added sugars. A possible explanation is households with more educated respondents (usually the female household head) may have a stronger preference for western food, and this may be indicative of the lifestyle changes that happen with economic development and increasing availability of ultra-processed foods due to westernization of food markets. For example, there is evidence that education might increase the demand for western food products purchased from modern markets [66], and frequent shopping at these markets is associated with higher consumption of ultra-processed foods for Vietnamese urban consumers [67].

Other household characteristics significantly associated with the share of food energy from added sugars include having children in the household that are less than five years of age, the presence of a cigarette smoker in the household, and city of residence (Hanoi). Individuals from households with children less than five years of age are more likely to consume a relatively lower share of their calories from added sugars. This supports findings from other studies in developing countries which show that household members’ diet quality tends to improve when there are young children present [56,57]. In contrast, individuals who live with any cigarette smoker in the household tend to have a higher share of calories from added sugars. Past studies have also found that households with smokers were more likely to have poorer diet quality, as smoking created an altered pattern of demand for some specific foods including high sugary food and beverages [68,69]. Lastly, individuals living in Hanoi are more likely to obtain a higher share of their calories from foods and beverages with added sugars as compared to those residing in Ho Chi Minh City. This could be due to some significant differences in eating habits, lifestyle, culture, and food availability found between the two cities [70].

### 3.2. Subsample Analysis

To explore the possible heterogeneous relationships between nutrition label use, concerns about sugar and the share of calories from added sugars, subsample analyses were conducted to compare males versus females for both adults and adolescents. The results are reported in Table 4 (for adults) and Table 5 (for adolescents). Overall, the empirical results show significant differences across gender for both the adult and adolescent analyses.

#### 3.2.1. Adults

As shown in Table 4, nutrition label use is shown to be negatively and significantly associated with the share of calories from added sugars for adult males only. Similar to the full sample analyses, the AvoidSugar variable is not statistically significant in explaining calorie share from added sugars for either male or female adults. Relative to younger adults (reference age group 19–29 years), older adults (both males and females) are more likely to consume less calories from foods with added sugars. This is possibly because they become more concerned about the healthfulness of their diet as they get older, as shown in other studies [56,60], and it may also suggest changing preferences for sugary foods (i.e., diet and nutrition transition) over time.

Similar to the full sample analysis reported in Table 2, the respondent’s education is positively associated with share of calories from foods with added sugars, however, the variable is only significant for male adults (Table 4). These results suggest that adult males’ food intake is likely to be influenced by the decisions of the main food shopper (the respondent), which in most cases was also the female head of the household.

#### 3.2.2. Adolescents

Table 5 reports the empirical results for adolescents. Interestingly, the two variables indicating that the main food shopper(the respondent) uses nutrition labels (LabelSugar) and avoids purchasing food and beverages high in sugar (AvoidSugar), both have negative, significant, and relatively large coefficients in the estimation of female adolescents’ share of calories from added sugars (SugarCalorie). The variable LabelSugar is also significant and large in the model for SugarCalorie. Other household characteristics show limited effects on the share of calories from added sugars. Again, the education of respondent and the city dummy variable (Hanoi) are shown to be positively correlated with calorie share from added sugars, but only for female adolescents.

Given that the highest share of calories from added sugars is observed among female (10.6% of calories) and male (9.9% of calories) adolescents (shown in Table 3), these results are useful to inform policymakers about the importance of providing access to nutrition information at the point of purchase and raising consumer awareness of the impacts of high consumption of added sugars. Use of nutrition labelling by the main food shopper for the household appears to significantly reduce the intake of added sugars for adolescents.

## 4. Conclusions

This study explores the interrelationships between nutrition label use, health concerns related to sugar consumption, and the share of caloric intake from food and beverages with added sugars among adults and adolescents in urban Vietnam. The empirical work is based on intra-household data from a comprehensive survey covering 4047 adults and 737 adolescents. We perform subsample analyses to test for the heterogenous associations of the explanatory variables on the share of calories from added sugars for adults and adolescents. The analysis for adolescents is especially interesting, given that adolescents may be at higher risk of overconsumption of added sugars than adults.

The empirical results consistently show that the use of sugar labels by the household’s main food shopper (respondent) is associated with a significantly lower share of caloric intake from added sugars. The magnitude of this association is relatively larger for adolescents as compared to adults. We also find that the level of education attained by household’s main food shopper is positively associated with calorie share from added sugars for individual’s living in the household.

Another important finding of this study is that main food shoppers’ reported attempts to avoid purchasing food and beverages with high amounts of added sugars are not significantly associated with the share of caloric intake from added sugars for adults. However, we find some evidence that attempts to avoid sugar by the household’s main food shopper can reduce female adolescents sugar consumption. This result is quite meaningful given that female adolescents are at greater risk of consuming a higher share of calories from added sugars, as compared to adults and male adolescents.

Considering these results, the Vietnamese government may want to be proactive and develop initiatives to tackle the impacts of increasing consumption of sugary foods and beverages. The government may want to prioritize increasing consumers’ awareness about the negative health consequences of consuming high amounts of added sugars and increase consumers access to information on nutrition profiles of food through food labeling or other point-of-purchase mechanisms. Early initiatives could include public health programs targeting female adolescents and the household food shoppers and food preparers (usually the mother). These programs could focus on increasing their nutrition and health knowledge, and improving awareness about the potential negative impacts of consuming food and beverages with added sugars, and increasing the use of nutrition labels on food and beverage products. Moreover, to achieve a large-scale reduction in added sugar consumption, food labels should include detailed, highly visible, and easily understood information about added sugars [38].

Our research contributes to the growing body of literature that explores the impact of nutrition label use on the dietary intake and quality. There are several limitations that future research might address. The cross-sectional nature of our data allows us to only examine associations between covariates and outcome variables, therefore we cannot make strong causal inferences. Additionally, our data cover only the two largest cities in Vietnam, Hanoi and Ho Chi Minh City, and therefore may not be fully representative of all Vietnamese urban households. Additional research on this topic should consider focusing on less developed urban, peri-urban, and rural areas of Vietnam, as well as other developing countries.

## Figures and Tables

**Table 1:** Descriptive statistics of variables included in the estimations.

Variables	Definition	Mean	Sd.	Min	Max
	**Dependent variable**				
SugarCalorie	Individual’s share of daily caloric intake from food and beverages with added sugars	6.88	6.86	0	44.76
	**Key explanatory variables**				
LabelSugar	1= Respondent uses nutrition labels to obtain information about sugar content in food products; 0= otherwise	0.38	0.48	0	1.00
AvoidSugar	1= Respondent avoids purchasing food and drinks with a high amount of sugar; 0= otherwise	0.73	0.44	0	1
	**Control variables**				
Age					
10–19 years	1= Individual’s age is between 10 and 19 years	0.15	0.36	0	1
19–29 years	1= Individual’s age is between 20 and 29 years	0.19	0.39	0	1
30–39 years	1= Individual’s age is between 30 and 39 years	0.23	0.42	0	1
40–49 years	1= Individual’s age is between 40 and 49 years	0.20	0.40	0	1
50–59 years	1= Individual’s age is between 50 and 59 years	0.14	0.35	0	1
60+ years	1= Individual’s age is 60+ years	0.09	0.29	0	1
Female	Gender of the individual (1= Female; 0= Male)	0.54	0.50	0	1
RespAge					
19–29 years	1= Respondent’s age between 20 and 29 years	0.14	0.35	0	1
30–39 years	1 = Respondent’s age between 30 and 39 years	0.28	0.45	0	1
40–49 years	1 = Respondent’s age between 40 and 49 years	0.32	0.47	0	1
50–59 years	1 = Respondent’s age between 50 and 59 years	0.20	0.40	0	1
60+ years	1 = Respondent’s age is 60+ years	0.06	0.24	0	1
RespEduc	Highest years of education completed by household respondent (years)	10.86	3.16	1	19
Income	Monthly household expenditure per capita (million VND per month)	2.51	1.36	0.43	29.65
Income^2^	Monthly household expenditure per capita (million VND per month), squared	8.16	25.53	0.18	878.9
Child_under5	1= Presence of at least 1 child aged below 5 years in the household; 0= No	0.30	0.46	0	1
Child_5to9	1= Presence of at least 1 child aged 5 to 9 years in the household; 0= No	0.26	0.44	0	1
Child_10to19	1= Presence of at least 1 child aged 10 to 19 years in the household; 0= No	0.46	0.50	0	1
Smoker	1= if any household member regularly smokes cigarettes; 0= No	0.54	0.50	0	1
Hanoi	1= Hanoi; 0= Ho Chi Minh City	0.41	0.49	0	1
	**Instrumental variables**				
DistanceWetMarket	Distance to the nearest wet market (km)	1.18	1.08	0.01	14
InfoSourceDoctor	1= If the source of nutrition information is from medical professionals; 0= No	0.68	0.47	0	1
HealthProblem	1= If any household member has any diet-related health problems; 0= No	0.14	0.35	0	1

VND/month represents Vietnamese dong per month. 1 USD = 22318 VND on 30 December 2016. *Source*: Authors’ estimation from Vietnam Urban Food Consumption and Expenditure Study. Sd= standard deviation.

**Table 2:** Three-stage least squares (3SLS) estimates for full sample. (n = 4784).

Variables	Sugar Calorie	Label Sugar	Avoid Sugar
AvoidSugar	0.565	0.169	
	(1.812)	(0.130)	
LabelSugar	−5.406 ***		−0.919 **
	(1.880)		(0.416)
Age (Ref: 10–19 years)			
20–29 years	−2.448 ***	−0.007	−0.048
	(0.397)	(0.028)	(0.035)
30–39 years	−3.830 ***	−0.007	−0.044
	(0.383)	(0.028)	(0.034)
40–49 years	−4.719 ***	0.003	0.008
	(0.343)	(0.024)	(0.031)
50–59 years	−4.545 ***	−0.000	−0.018
	(0.410)	(0.029)	(0.037)
≥60 years	−4.600 ***	−0.062 **	−0.087 *
	(0.461)	(0.031)	(0.053)
Female	3.047 ***	0.009	0.006
	(0.193)	(0.014)	(0.018)
RespAge (Ref: 21–29 years)			
30–39 years	0.263	−0.008	−0.012
	(0.374)	(0.026)	(0.034)
40–49 years	0.880 **	0.012	−0.042
	(0.388)	(0.027)	(0.034)
50–59 years	0.753 *	0.001	−0.048
	(0.400)	(0.028)	(0.037)
≥60 years	−0.572	0.000	−0.154 ***
	(0.575)	(0.041)	(0.050)
RespEduc	0.171 ***	0.021 ***	0.024 **
	(0.056)	(0.003)	(0.010)
Income	−0.126	−0.025 **	−0.085 ***
	(0.165)	(0.011)	(0.019)
Income^2^	−0.003	0.000	0.003 ***
	(0.007)	(0.001)	(0.001)
Child_under5	−0.526 **	−0.016	−0.057 **
	(0.253)	(0.018)	(0.024)
Child_5to9	0.352	0.021	0.022
	(0.242)	(0.017)	(0.023)
Child_10to19	0.255	0.034 *	0.006
	(0.257)	(0.018)	(0.026)
Smoker	0.781 ***	0.035 **	0.015
	(0.207)	(0.014)	(0.021)
Hanoi	0.831 **	0.175 ***	0.130 *
	(0.406)	(0.016)	(0.075)
DistanceWetMarket		−0.021 ***	
		(0.006)	
InfoSourceDoctor			0.070 **
			(0.030)
HealthProblem			0.154 ***
			(0.052)
Constant	7.390 ***	0.003	0.948 ***
	(1.750)	(0.124)	(0.091)

Standard errors are in parentheses (*** *p* < 0.01, ** *p* < 0.05, * *p* < 0.1). *n*, number of observations.

**Table 3:** Share of caloric intake from food and beverages with added sugars, by gender and age groups.

Age Groups	Male		Female	
	*N*	Mean (Sd.)	*N*	Mean (Sd.)
10–19 years	377	9.91 (7.45)	360	10.56 (7.36)
20–29 years	383	5.70 (5.89)	508	9.21 (7.52)
30–39 years	508	3.96 (5.11)	592	8.07 (6.94)
40–49 years	455	3.86 (4.99)	481	7.30 (6.42)
50–59 years	317	4.05 (5.36)	372	7.23 (7.19)
≥ 60 years	174	4.85 (6.38)	257	6.39 (6.35)

*N*, number of observations and Sd., standard deviation.

**Table 4:** 3SLS estimates for adults.

Variables	Male Adults (≥20 Years) (*n* = 1837)			Female Adults (≥20 Years) (*n* = 2210)		
	Sugar Calorie	Label Sugar	Avoid Sugar	Sugar Calorie	Label Sugar	Avoid Sugar
AvoidSugar	−0.984	0.452 *		1.671	0.160	
	(2.887)	(0.258)		(2.563)	(0.172)	
LabelSugar	−5.630**		−0.706	−2.207		−0.805
	(2.437)		(0.495)	(3.073)		(0.567)
AgeGroup (Ref: 20–29 years)						
30–39 years	−1.501 ***	−0.005	−0.031	−0.881	−0.004	0.025
	(0.439)	(0.038)	(0.045)	(0.650)	(0.043)	(0.053)
40–49 years	−1.901 ***	0.011	0.054	−2.700 ***	0.001	0.033
	(0.452)	(0.039)	(0.045)	(0.595)	(0.040)	(0.048)
50–59 years	−1.966 ***	−0.004	0.012	−2.394 ***	0.013	0.043
	(0.440)	(0.038)	(0.044)	(0.582)	(0.039)	(0.047)
≥60 years	−0.665	−0.037	−0.029	−2.856 ***	−0.087 **	−0.045
	(0.545)	(0.047)	(0.061)	(0.679)	(0.042)	(0.078)
Female	−1.501 ***	−0.005	−0.031	−0.881	−0.004	0.025
	(0.439)	(0.038)	(0.045)	(0.650)	(0.043)	(0.053)
RespAge (Ref: 21–29 years)						
30–39 years	−0.044	−0.012	−0.015	−0.079	−0.003	−0.020
	(0.466)	(0.040)	(0.047)	(0.692)	(0.046)	(0.056)
40–49 years	0.766	−0.001	−0.074	1.174 *	0.010	−0.027
	(0.526)	(0.046)	(0.052)	(0.649)	(0.043)	(0.053)
50–59 years	0.689	−0.016	−0.094	0.983	0.010	−0.025
	(0.535)	(0.046)	(0.058)	(0.638)	(0.043)	(0.052)
≥60 years	−0.966	−0.002	−0.142 *	−0.517	0.042	−0.153 **
	(0.721)	(0.063)	(0.075)	(0.938)	(0.062)	(0.067)
RespEduc	0.175 **	0.017 ***	0.018	0.092	0.021 ***	0.023*
	(0.070)	(0.005)	(0.011)	(0.090)	(0.004)	(0.014)
Income	−0.328	−0.013	−0.076 ***	0.160	−0.027 *	−0.092 ***
	(0.224)	(0.019)	(0.026)	(0.260)	(0.016)	(0.028)
Income^2^	0.008	−0.000	0.003 ***	−0.013	0.001	0.003 ***
	(0.011)	(0.001)	(0.001)	(0.011)	(0.001)	(0.001)
Child_under5	−0.328	−0.023	−0.069 *	−0.607	−0.010	−0.043
	(0.357)	(0.030)	(0.040)	(0.378)	(0.025)	(0.031)
Child_5to9	0.361	0.034	0.041	0.472	0.023	0.027
	(0.344)	(0.029)	(0.039)	(0.379)	(0.025)	(0.033)
Child_10to19	0.431	0.028	0.000	−0.044	0.047 *	0.008
	(0.332)	(0.028)	(0.035)	(0.385)	(0.024)	(0.039)
Smoker	0.827 ***	0.026	0.006	0.781 **	0.034 *	0.010
	(0.277)	(0.023)	(0.029)	(0.322)	(0.020)	(0.029)
Hanoi	1.341 **	0.195 ***	0.105	−0.065	0.158 ***	0.080
	(0.575)	(0.026)	(0.099)	(0.614)	(0.024)	(0.093)
Constant	5.848 **	−0.179	0.943 ***	6.930 ***	0.015	0.878 ***
	(2.539)	(0.227)	(0.134)	(2.331)	(0.156)	(0.113)

Standard errors are in parentheses (*** *p* < 0.01, ** *p* < 0.05, * *p* < 0.1). *n*, number of observations.

**Table 5:** 3SLS estimates for adolescents.

Variables	Male Adolescents (11–19 Years) (*n* = 377)			Female Adolescents (11–19 Years) (*n* = 360)		
	Sugar Calorie	Label Sugar	Avoid Sugar	Sugar Calorie	Label Sugar	Avoid Sugar
AvoidSugar	27.474	0.594		−18.951 **	−0.451	
	(17.167)	(0.752)		(7.639)	(0.304)	
LabelSugar	−20.698 *		0.516	−28.394***		−0.928
	(10.834)		(1.163)	(8.095)		(0.698)
RespAge (Ref: 21–29 years)						
30–39 years	1.822	0.005	−0.025	−1.741	−0.042	−0.065
	(3.061)	(0.143)	(0.161)	(3.312)	(0.149)	(0.152)
40–49 years	4.910	0.086	−0.117	−1.253	0.025	−0.005
	(3.446)	(0.154)	(0.159)	(3.200)	(0.145)	(0.150)
50–59 years	4.809	0.125	−0.163	−3.192	−0.022	−0.035
	(4.151)	(0.182)	(0.184)	(3.668)	(0.166)	(0.171)
≥60 years	5.890	−0.076	−0.166	−2.119	−0.067	−0.074
	(5.863)	(0.282)	(0.390)	(4.753)	(0.214)	(0.239)
RespEduc	0.588 *	0.022*	−0.011	0.691 **	0.026 **	0.020
	(0.345)	(0.012)	(0.031)	(0.347)	(0.013)	(0.023)
Income	2.665	0.008	−0.040	−0.340	−0.049	−0.127
	(1.680)	(0.081)	(0.078)	(2.682)	(0.120)	(0.114)
Income^2^	−0.192	0.002	0.001	−0.298	0.001	0.014
	(0.191)	(0.009)	(0.010)	(0.435)	(0.020)	(0.020)
Child_under5	0.796	0.010	−0.031	−2.206	−0.019	−0.052
	(1.764)	(0.083)	(0.089)	(1.684)	(0.076)	(0.075)
Child_5to9	2.360	−0.023	−0.030	−2.574 *	−0.019	−0.035
	(1.751)	(0.084)	(0.102)	(1.432)	(0.064)	(0.065)
Smoker	4.598 *	0.169 **	−0.142	1.046	0.006	0.010
	(2.507)	(0.075)	(0.152)	(1.199)	(0.054)	(0.055)
Hanoi	3.305	0.181 ***	−0.110	7.509 ***	0.243 ***	0.263
	(2.389)	(0.067)	(0.188)	(2.537)	(0.071)	(0.176)
Constant	−21.250	−0.530	0.949 ***	30.405 ***	0.494	0.968 ***
	(17.033)	(0.756)	(0.267)	(9.377)	(0.386)	(0.251)

Standard errors in parentheses (*** *p* < 0.01, ** *p* < 0.05, * *p* < 0.1). *n*, number of observations.

## Appendix A

**Table A1:** List of food and beverages with added sugars.

English Name	Vietnamese Name
Sweet sticky rice	Xôi ngọt có đường
Sweet soup with sticky rice	Xôi chè
Vietnamese hollow doughnuts	Bánh tiêu
Sweet Popcorn	Bỏng ngô có đường/mật
Floating cake (dessert wading in water)	Bánh trôi nước
Sweet sticky rice cake	Bánh nếp (ngọt)
Sponge cake	Bánh bò (bột gạo nếp)
Sweet puffed rice	Bỏng gạo có đường
Glutinous rice cake	Bánh gai
Donuts (Vietnamese style)	Bánh cam (bánh rán)
Corn sweet soup	Chè ngô
Young rice cake	Bánh cốm
Chè mixed beans (many types of bean)	Chè thập cẩm
Tau Pho (tofu syrup)	Tào phớ (đậu hủ nước đường)
Green bean sweet soup	Chè đỗ xanh
Peanut candy	Kẹo lạc
Black bean sweet soup	Chè đỗ đen
Black sesame milk	Sữa mè đen
Green bean cake	Bánh đậu xanh
Black bean milk	Sữa đậu đen
Dried jackfruit chips	Mít sấy
Fried banana cake	Chuối chiên
Banana ice cream	Kem chuối
Sweet fruit mixture	Trái cây dầm
Drinking yogurt	Sữa chua uống
Soya milk	Sữa đậu nành có đường
Pasteurized milk	Sữa tiệt trùng
Sweet yogurt	Sữa chua có đường
Sweetened condensed milk	Sữa đặc có đường
Cream	Kem
Glutinous rice yogurt	Sữa chua nếp
Fresh fruit juice	Nước ép từ trái cây tươi
Carbonated soft drink (e.g., Coke, Pepsi)	Nước ngọt có ga (v.d. Coca Cola, Pepsi)
Instant coffee (3-in−1 with sugar and cream)	Cà phê hòa tan
Bottled green tea	Trà xanh đóng chai
Energy drink (e.g., Sting, Red bull)	Nước tăng lực (v.d. Sting, Red Bull)
Milk tea	Trà sữa
Lipton tea bag	Trà túi lọc Lipton
Smoothie (e.g., avocado, strawberry)	Sinh tố (v.d. bơ, dâu)
Sugarcane juice	Nước mía
Lotus seed sweet soup	Chè hạt sen
Fruit mix sweet soup (e.g., mango, pomelo)	Chè trái cây (v.d. chè xoài, chè bưởi)
Banana sweet soup	Chè chuối
Bottled herbal tea	Trà thảo mộc đóng chai
Fruit milk/ Corn milk	Sữa trái cây/ Sữa ngô
Bird nest drink	Nước yến
Lemon tea	Trà chanh
Lotus seed milk	Sữa hạt sen
Hot cocoa liquid, with added sugar	Đồ uống ca cao thêm đường
Cake in general	Bánh ngọt không nêu rõ
Chewing gum	Kẹo cao su
Choco pie cake	Bánh Chocopie
Lollipop / candy	Kẹo mút / Kẹo
Chicken thighs cake	Bánh đùi gà
Egg cake (e.g., Custas cake)	Bánh ngọt nhân trứng (Bánh Custas)
Banana cake	Bánh chuối
Biscuits	Bánh qui
Rice cake (sweet)	Bánh gạo ngọt
AFC cake	Bánh AFC
Birthday cake/Cheesecake	Bánh kem sinh nhật
Sweet jelly	Thạch rau câu ngọt
Cream puffs	Bánh su kem
Caramel/ Flan	Caramen / Bánh Flan
Chocolate	Kẹo Sô-cô-la
Pig skin cakes	Bánh da lợn
“Chè lam” cake	Bánh chè lam
Coconut jams	Mứt dừa
Theochew pastry	Bánh pía
Fried sweet potato cake	Bánh khoai
Cassava sweet soup	Chè sắn
Taro sweet soup	Chè khoai môn

## References

[1] Afshin A., Sur P.J., Fay K.A., Cornaby L., Ferrara G., Salama J.S., Mullany E.C., Abate K.H., Abbafati C., Abebe Z. (2019). Health effects of dietary risks in 195 countries, 1990–2017: A systematic analysis for the global burden of disease study 2017. Lancet.

[2] Gaziano T.A., Bitton A., Anand S., Abrahams-Gessel S., Murphy A. (2010). Growing epidemic of coronary heart disease in low- and middle-income countries. Curr. Probl. Cardiol..

[3] Mozaffarian D. (2016). Dietary and policy priorities for cardiovascular disease, diabetes, and obesity. Circulation.

[4] Harris J., Nguyen P.H., Tran L.M., Huynh P.N. (2020). Nutrition transition in Vietnam: Changing food supply, food prices, household expenditure, diet and nutrition outcomes. Food Secur..

[5] Popkin B.M., Corvalan C., Grummer-Strawn L.M. (2020). Dynamics of the double burden of malnutrition and the changing nutrition reality. Lancet.

[6] Aurino E., Fernandes M., Penny M.E. (2017). The nutrition transition and adolescents’ diets in low- and middle-income countries: A cross-cohort comparison. Public Health Nutr..

[7] Drewnowski A., Tappy L., Forde C.G., McCrickerd K., Tee E.S., Chan P., Amin L., Trinidad T.P., Amarra M.S. (2019). Sugars and sweeteners: Science, innovations, and consumer guidance for Asia. Asia Pac. J. Clin. Nutr..

[8] Umberger W.J., He X., Minot N., Toiba H. (2015). Examining the relationship between the use of supermarkets and over-nutrition in Indonesia. Am. J. Agric. Econ..

[9] Kelly M., Jackson P., Spiess W.E.L., Sultana F. (2016). The nutrition transition in developing Asia: Dietary change, drivers and health impacts. Eating, Drinking: Surviving: The International Year of Global Understanding—IYGU.

[10] Sigman-Grant M., Morita J. (2003). Defining and interpreting intakes of sugars. Am. J. Clin. Nutr..

[11] Bailey R.L., Fulgoni V.L., Cowan A.E., Gaine P.C. (2018). Sources of added sugars in young children, adolescents, and adults with low and high intakes of added sugars. Nutrients.

[12] Vartanian L.R., Schwartz M.B., Brownell K.D. (2007). Effects of soft drink consumption on nutrition and health: A systematic review and meta-analysis. Am. J. Public Health.

[13] Khan T.A., Sievenpiper J.L. (2016). Controversies about sugars: Results from systematic reviews and meta-analyses on obesity, cardiometabolic disease and diabetes. Eur. J. Nutr..

[14] Rippe J.M., Angelopoulos T.J. (2016). Relationship between added sugars consumption and chronic disease risk factors: Current understanding. Nutrients.

[15] Nguyen T.T., Hoang M.V. (2018). Non-communicable diseases, food and nutrition in Vietnam from 1975 to 2015: The burden and national response. Asia Pac. J. Clin. Nutr..

[16] Luong L., Vu L.H. (2020). Impacts of excise taxation on non-alcoholic beverage consumption in Vietnam. Sustainability.

[17] The Department of Preventive Medicine Vietnam (2017). Actual Situation of Disease Burden and the Orientation of Non-Communicable Diseases Prevention and Management in the Coming Period.

[18] World Health Organisation (2015). Guideline: Sugars Intake for Adults and Children. https://www.who.int/publications/i/item/9789241549028.

[19] Micha R., Mannar V., Afshin A., Allemandi L., Baker P., Battersby J., Bhutta Z., Chen K., Corvalan C., Di Cesare M. (2020). 2020 Global Nutrition Report. 2020 Global Nutrition Report: Action on Equity to End Malnutrition.

[20] Nguyen P.V.N., Hong T.K., Hoang T., Nguyen D.T., Robert A.R. (2013). High prevalence of overweight among adolescents in Ho Chi Minh City, Vietnam. BMC Public Health.

[21] Beal T., Le T.D., Trinh H.T., Burra D.D., Béné C., Huynh T.T.T., Truong M.T., Nguyen S.D., Tran D.T., Nguyen K.T. (2020). Child overweight or obesity is associated with modifiable and geographic factors in Vietnam: Implications for program design and targeting. Nutrients.

[22] Miyakawa M., Shimizu T., Van Dat N., Thanh P., Thuy P.T.P., Anh N.T.H., Chau N.H., Matsushita Y., Kajio H., Mai V.Q. (2017). Prevalence, perception and factors associated with diabetes mellitus among the adult population in central Vietnam: A population-based, cross-sectional seroepidemiological survey. BMC Public Health.

[23] World Health Organisation Diabetes Country Profiles, 2016—Vietnam. https://www.who.int/diabetes/country-profiles/vnm_en.pdf?ua=1.

[24] Le N.T.D., Dinh Pham L., Quang Vo T. (2017). Type 2 diabetes in Vietnam: A cross-sectional, prevalence-based cost-of-illness study. Diabetes Metab. Syndr. Obes..

[25] United States Department of Agriculture, Foreign Agricultural Service (USDA FAS) (2017). Vietnam: GVN Revised Decree on Goods Labeling. Global Agricultural Information Network (GAIN) Report VM7031. https://www.fas.usda.gov/data/vietnam-gvn-revised-decree-goods-labeling.

[26] Cowburn G., Stockley L. (2005). Consumer understanding and use of nutrition labelling: A systematic review. Public Health Nutr..

[27] Ikonen I., Sotgiu F., Aydinli A., Verlegh P.W.J. (2020). Consumer effects of front-of-package nutrition labeling: An interdisciplinary meta-analysis. J. Acad. Mark. Sci..

[28] Anastasiou K., Miller M., Dickinson K. (2019). The relationship between food label use and dietary intake in adults: A systematic review. Appetite.

[29] Miller L.M.S., Cassady D.L., Applegate E.A., Beckett L.A., Wilson M.D., Gibson T.N., Ellwood K. (2015). Relationships among food label use, motivation, and dietary quality. Nutrients.

[30] Kim S.-Y., Nayga R.M., Capps O. (2001). Food label use, self-selectivity, and diet quality. J. Consum. Aff..

[31] Pérez-Escamilla R., Haldeman L. (2002). Food label use modifies association of income with dietary quality. J. Nutr..

[32] Drichoutis A.C., Lazaridis P., Nayga R.M. (2006). Consumers’ use of nutritional labels: A review of research studies and issues. Acad. Mark. Sci. Rev..

[33] Variyam J.N. (2008). Do nutrition labels improve dietary outcomes?. Health Econ..

[34] Neuhouser M.L., Kristal A.R., Patterson R.E. (1999). Use of food nutrition labels is associated with lower fat intake. J. Am. Diet. Assoc..

[35] Kreuter M.W., Brennan L.K., Scharff D.P., Lukwago S.N. (1997). Do nutrition label readers eat healthier diets? Behavioral correlates of adults’ use of food labels. Am. J. Prev. Med..

[36] Ollberding N.J., Wolf R.L., Contento I. (2010). Food label use and its relation to dietary intake among us adults. J. Am. Diet. Assoc..

[37] Kim H.-S., Oh C., No J.-K. (2016). Can nutrition label recognition or usage affect nutrition intake according to age?. Nutrition.

[38] Weaver D., Finke M. (2003). The relationship between the use of sugar content information on nutrition labels and the consumption of added sugars. Food Policy.

[39] Barreiro-Hurlé J., Gracia A., de-Magistris T. (2010). Does nutrition information on food products lead to healthier food choices?. Food Policy.

[40] Grimes C.A., Riddell L.J., Nowson C.A. (2009). Consumer knowledge and attitudes to salt intake and labelled salt information. Appetite.

[41] Hoefkens C., Verbeke W., Van Camp J. (2011). European consumers’ perceived importance of qualifying and disqualifying nutrients in food choices. Food Qual. Prefer..

[42] Kim S.-Y., Nayga R.M., Capps O. (2000). The effect of food label use on nutrient intakes: An endogenous switching regression analysis. J. Agric. Resour. Econ..

[43] Fisberg M., Kovalskys I., Gómez G., Rigotti A., Sanabria L.Y.C., García M.C.Y., Torres R.G.P., Herrera-Cuenca M., Zimberg I.Z., Koletzko B. (2018). Total and added sugar intake: Assessment in eight latin american countries. Nutrients.

[44] Toiba H., Umberger W.J., Minot N. (2015). Diet transition and supermarket shopping behaviour: Is there a link?. Bull. Indones. Econ. Stud..

[45] Rupa J.A., Umberger W.J., Zeng D. (2019). Does food market modernisation lead to improved dietary diversity and diet quality for urban Vietnamese households?. Aust. J. Agric. Resour. Econ..

[46] Salois M.J., Tiffin R., Balcombe K.G. (2012). Impact of income on nutrient intakes: Implications for undernourishment and obesity. J. Dev. Stud..

[47] Trinh T.H., Simioni M., Thomas-Agnan C. (2018). Assessing the nonlinearity of the calorie-income relationship: An estimation strategy—With new insights on nutritional transition in Vietnam. World Dev..

[48] Jezewska-Zychowicz M., Wadolowska L., Kowalkowska J., Lonnie M., Czarnocinska J., Babicz-Zielinska E. (2017). Perceived health and nutrition concerns as predictors of dietary patterns among Polish females aged 13–21 years (gebahealth project). Nutrients.

[49] Sun Y.-H.C. (2008). Health concern, food choice motives, and attitudes toward healthy eating: The mediating role of food choice motives. Appetite.

[50] Umberger W.J., Rupa J.A., Zeng D. (2020). Understanding food westernisation and other contemporary drivers of adult, adolescent and child nutrition quality in urban Vietnam. Public Health Nutr..

[51] Charrondiere U., Haytowitz D., Stadlmayr B. (2012). FAO/INFOODS density database, version 2.0. Food and Agriculture Organization (FAO) of the United Nations (UN) Technical Workshop Report.

[52] National Institute of Nutrition, M.o.H (2017). Vietnamese Food Composition Table.

[53] Gibson R.S., Ferguson E.L. (2008). An Interactive 24-Hour Recall for Assessing the Adequacy of Iron and Zinc Intakes in Developing Countries.

[54] MacLean W., Harnly J., Chen J., Chevassus-Agnes S., Gilani G., Livesey G., Warwick P. (2003). Food energy-methods of analysis and conversion factors. Food and Agriculture Organization of the United Nations Technical Workshop Report.

[55] Huynh D.T.T., Dibley M.J., Sibbritt D.W., Tran H.T.M. (2008). Energy and macronutrient intakes in preschool children in urban areas of Ho Chi Minh City, Vietnam. BMC Pediatrics.

[56] Amarra M.S.V., Khor G.L., Chan P. (2016). Intake of added sugar in malaysia: A review. Asia Pac. J. Clin. Nutr..

[57] Ha K., Chung S., Lee H.-S., Kim C.-i., Joung H., Paik H.-Y., Song Y. (2016). Association of dietary sugars and sugar-sweetened beverage intake with obesity in korean children and adolescents. Nutrients.

[58] Azaïs-Braesco V., Sluik D., Maillot M., Kok F., Moreno L.A. (2017). A review of total and added sugar intakes and dietary sources in Europe. Nutr. J..

[59] Lin B.-H., Yen S.T. (2008). Consumer knowledge, food label use and grain consumption in the US. Appl. Econ..

[60] Schroeter C., Anders S., Carlson A. (2012). The economics of health and vitamin consumption. Appl. Econ. Perspect. Policy.

[61] Zellner A., Theil H., Raj B., Koerts J. (1992). Three-stage least squares: Simultaneous estimation of simultaneous equations. Henri Theil’s Contributions to Economics and Econometrics: Econometric Theory and Methodology.

[62] Van Dillen S.M., Hiddink G.J., Koelen M.A., de Graaf C., van Woerkum C.M. (2003). Understanding nutrition communication between health professionals and consumers: Development of a model for nutrition awareness based on qualitative consumer research. Am. J. Clin. Nutr..

[63] Wertheim-Heck S.C.O., Vellema S., Spaargaren G. (2015). Food safety and urban food markets in Vietnam: The need for flexible and customized retail modernization policies. Food Policy.

[64] Antonakis J., Bendahan S., Jacquart P., Lalive R. (2010). On making causal claims: A review and recommendations. Leadersh. Q..

[65] Staiger D., Stock J.H. (1997). Instrumental variables regression with weak instruments. Econometrica.

[66] Mergenthaler M., Weinberger K., Qaim M. (2009). The food system transformation in developing countries: A disaggregate demand analysis for fruits and vegetables in Vietnam. Food Policy.

[67] Wertheim-Heck S.C.O., Raneri J.E., Oosterveer P. (2019). Food safety and nutrition for low-income urbanites: Exploring a social justice dilemma in consumption policy. Environ. Urban..

[68] Margetts B.M., Jackson A.A. (1993). Interactions between people’s diet and their smoking habits: The dietary and nutritional survey of British adults. Br. Med. J..

[69] Dallongeville J., Marécaux N., Fruchart J.-C., Amouyel P. (1998). Cigarette smoking is associated with unhealthy patterns of nutrient intake: A meta-analysis. J. Nutr..

[70] Van Dinh T., Van Dong H., Thanh Chung N., Lee A.H. (2013). Validity and reliability of a food frequency questionnaire to assess habitual dietary intake in northern Vietnam. Vietnam J. Public Health.

